# A new species of *Burkholderia* isolated from sugarcane roots promotes plant growth

**DOI:** 10.1111/1751-7915.12105

**Published:** 2013-12-19

**Authors:** Chanyarat Paungfoo-Lonhienne, Thierry G A Lonhienne, Yun Kit Yeoh, Richard I Webb, Prakash Lakshmanan, Cheong Xin Chan, Phaik-Eem Lim, Mark A Ragan, Susanne Schmidt, Philip Hugenholtz

**Affiliations:** 1 School of Agriculture and Food Sciences, The University of QueenslandSt. Lucia, Qld, 4072, Australia; 2 Institute for Molecular Bioscience, The University of QueenslandSt. Lucia, Qld, 4072, Australia; 3 ARC Centre of Excellence in Bioinformatics, The University of QueenslandSt. Lucia, Qld, 4072, Australia; 4 School of Chemistry and Molecular Biosciences, The University of QueenslandSt. Lucia, Qld, 4072, Australia; 5 Australian Centre for Ecogenomics, School of Chemistry and Molecular Biosciences, The University of QueenslandSt. Lucia, Qld, 4072, Australia; 6 Centre for Microscopy and Microanalysis, The University of QueenslandSt. Lucia, Qld, 4072, Australia; 7 Sugar Research AustraliaIndooroopilly, Qld, 4068, Australia; 8 Institute of Biological Sciences and Institute of Ocean and Earth Sciences, University of Malaya50603, Kuala Lumpur, Malaysia

## Abstract

Sugarcane is a globally important food, biofuel and biomaterials crop. High nitrogen (N) fertilizer rates aimed at increasing yield often result in environmental damage because of excess and inefficient application. Inoculation with diazotrophic bacteria is an attractive option for reducing N fertilizer needs. However, the efficacy of bacterial inoculants is variable, and their effective formulation remains a knowledge frontier. Here, we take a new approach to investigating diazotrophic bacteria associated with roots using culture-independent microbial community profiling of a commercial sugarcane variety (Q208^A^) in a field setting. We first identified bacteria that were markedly enriched in the rhizosphere to guide isolation and then tested putative diazotrophs for the ability to colonize axenic sugarcane plantlets (Q208^A^) and promote growth in suboptimal N supply. One isolate readily colonized roots, fixed N_2_ and stimulated growth of plantlets, and was classified as a new species, *Burkholderia australis* sp. nov. Draft genome sequencing of the isolate confirmed the presence of nitrogen fixation. We propose that culture-independent identification and isolation of bacteria that are enriched in rhizosphere and roots, followed by systematic testing and confirming their growth-promoting capacity, is a necessary step towards designing effective microbial inoculants.

## Introduction

Sugarcane (*Saccharum officinarum* x *spontaneum* L.) is one of the most important agricultural crops globally and a source of sugar, renewable energy and biomaterials. Sugarcane is grown in over 110 tropical and subtropical countries with 50% of global production generated in Brazil and India (Fischer *et al*., 2012). Although sugarcane is a carbon crop (sugar and biomass are harvested rather than protein-rich grains), high rates of nitrogen (N) fertilizer are often applied to maximize yields. High cost for N fertilizers and off-site N losses are a problem, as are declining sugarcane yields despite high agronomic input (Bell *et al*., 2007; Brodie *et al*., 2008). While N fertilizer use is comparatively low in Brazil (∼50 kg N ha^−1^), rates in other producer countries average ∼120 to 300 kg N ha^−1^ with extreme rates in excess of 700 kg N ha^−1^, and the weighted global average indicating that only 50% of N fertilizer is used by crops (Robinson *et al*., 2011). Reasons for the low fertilizer use efficiency include high soil nitrification rates and weather extremes that promote N leaching and denitrification (Robinson *et al*., 2011). In addition, manufacture of synthetic N fertilizer uses ≈ 2% of global energy consumption.

One avenue to remediate the problem associated with synthetic N fertilizers is the use of microbes capable of biological N_2_ fixation (BNF) (de Carvalho *et al*., 2011). There is evidence that endophytic bacteria able to fix N_2_ contribute to the N budget of Brazilian sugarcane (Boddey *et al*., 2003; Urquiaga *et al*., 2012). Diazotrophic bacteria belonging to the genera *Gluconacetobacter* (Cavalcante and Dobereiner, 1988), *Azospirillum* (Baldani *et al*., 1997), *Burkholderia* (Govindarajan *et al*., 2006) and *Herbaspirillum* (Baldani *et al*., 1996) have been isolated from intercellular spaces, roots and rhizosphere of sugarcane (James and Olivares, 1998; James, 2000; Fischer *et al*., 2012), although it remains challenging to quantify the contribution of BNF to the crop N budget. The efficacy of diazotrophic bacteria varies considerably in sugarcane crops (Yoneyama *et al*., 1997). Further, rhizosphere bacteria (Smalla *et al*., 2001; İnceoğlu *et al*., 2012) including diazotrophic bacteria (Shrestha and Ladha, 1996; Sasaki *et al*., 2010) have cultivar-specific relationships, and sugarcane varieties may have specific controls aimed at attracting specific diazotrophs (de Carvalho *et al*., 2011). Overall, inconsistent responses of crop cultivars and growth locations limit the success of ‘biofertilizers’ based on diazotrophic and, otherwise, plant growth-promoting rhizobacteria (PGPR) (Figueiredo *et al*., 2011).

These considerations confirm the need for new approaches that lead to successful associations between diazotrophic bacteria and sugarcane roots. Our research focussed on Australian sugarcane because breeding and cropping occurs in the presence of relatively high N fertilizer rates (Whan *et al*., 2010) that may have selected against BNF associations. We aimed to use culture-independent community profiling as a novel approach to identify the most abundant root/rizhosphere microbes and search for potential diazotrophs, and to better understand root and rhizosphere microbial assemblages and their functional association with sugarcane in relation to N nutrition and growth. In that process, three genera of putative diazotrophic bacteria that were abundant in rhizosphere/root were isolated and tested for their ability to promote growth in sugarcane. One isolate was able to stimulate growth of sugarcane plantlets in suboptimal N supply and was characterized functionally and taxonomically.

## Results and discussion

### Identification of bacterial diazotrophs associated with sugarcane variety Q208^A^

Our first step was to characterize bacterial communities of the combined rhizosphere and roots of Q208^A^, a high-yielding and widely grown sugarcane variety in Australia, followed by identification of putative bacterial diazotrophs. We used 16S rRNA gene amplicon pyrosequencing and compared microbial community profiles between rhizosphere + root and bulk soil. DNA isolated from these biological samples was sequenced using primers broadly targeting bacterial and archaeal 16S rRNA genes. The sequences so obtained were grouped into operational taxonomic units (OTUs) and classified against the greengenes taxonomy (DeSantis *et al*., 2006) providing genus-level resolution of communities. The relative abundance of identified OTUs was compared between bulk soil and rhizosphere/root, and ranked by fold enrichment to highlight potential plant growth-promoting populations including those capable of BNF (Table 1). BNF associated with sugarcane in other countries (notably Brazil, India and Uruguay) including *Azospirillum*, *Azotobacter*, *Gluconacetobacter* and *Herbaspirillum* (Baldani *et al*., 1997; Boddey *et al*., 2003) were not detected in the rhizosphere + root of Q208^A^. This suggests that plant-associated BNF may be specific to particular geographical regions (Yoneyama *et al*., 1997) and/or that Australian sugarcane soils harbour a different suite of bacteria.

**Table 1:** The 20 most abundant bacterial clusters enriched in the rhizosphere + root of sugarcane Q208^A^

	Soil	Rhizosphere and Root		
OTU	Relative abundance (%)		Fold enrichment	Genus identification (Phylum)
OTU 26	0.12	2.38	19.9	*Streptomyces* (Actinobacteria)
**OTU 2**	1.18	20.76	**17.5**	***Bacillus* (Firmicutes)**
OTU 267	0.02	0.30	16.1	*Microbispora* (Actinobacteria)
OTU 17	0.09	1.16	12.9	*Micrococcus* (Actinobacteria)
OTU 93	0.05	0.55	11.7	*Terrabacter* (Actinobacteria)
**OTU 11**	0.05	0.52	**10.1**	***Burkholderia* (Proteobacteria)**
OTU 10	0.13	1.25	9.7	*Actinoplanes* (Actinobacteria)
OTU 73	0.07	0.63	9.5	*Leifsonia* (Actinobacteria)
OTU 47	0.09	0.80	8.8	*Blastococcus* (Actinobacteria)
**OTU 16**	0.21	1.44	**6.8**	***Rhizobium* (Proteobacteria)**
OTU 162	0.06	0.31	5.1	*Ammoniphilus* (Firmicutes)
OTU 48	0.57	2.72	4.8	*Actinoplanes* (Actinobacteria)
**OTU 8**	0.42	1.85	**4.4**	***Bacillus* (Firmicutes)**
OTU 13	0.16	0.55	3.3	*Paludibacterium* (Proteobacteria)
**OTU 27**	0.19	0.44	**2.4**	***Burkholderia* (Proteobacteria)**
OTU 161	0.33	0.72	2.2	Unclassified (Chloroflexi)
OTU 357	0.21	0.43	2.1	Unclassified Syntrophobacteraceae (Proteobacteria)
OTU 34	0.46	0.84	1.8	Unclassified Chitinophagaceae (Bacteroidetes)
OTU 92	0.40	0.62	1.6	*Solirubrobacter* (Actinobacteria)
OTU 15	1.93	2.22	1.2	*Aquabacterium* (Proteobacteria)

Fold enrichment denotes the abundance of clusters in rhizosphere + root compared with soil. Clusters in bold were the subject of this study.

Among the 20 most-enriched OTUs in sugarcane rhizosphere + root, three OTUs belong to genera that contain known diazotrophic representatives, *Bacillus* (OTU 2&8), *Burkholderia* (OTU 11&27) and *Rhizobium* (OTU 16; Table 1). *Bacillus rhizosphaerae* has the ability for BNF (Madhaiyan *et al*., 2011), and so do many members of the genus *Burkholderia* (Suárez-Moreno *et al*., 2012). *Rhizobium* is a genus that contains many BNF symbionts of legumes (Denison and Kiers, 2004) and species with plant growth-promoting properties when associated with non-legumes (Chi *et al*., 2005). We aimed to isolate bacteria corresponding to these OTUs because of their high enrichment (up to 17.5-fold) in rhizosphere + root and potential for BNF (Table 1).

### *Burkholderia* isolate promotes growth of sugarcane plantlets

Primers specific to sequences of OTUs 2&8, 11&27 and 16 were designed for PCR screening of bacterial isolates. The bacterial pool of the rhizosphere + root samples was grown on R2A minimal medium and discrete colonies were screened for one of the three OTUs. Positive single colonies were obtained for the three primer sets, and the near full-length 16S rRNA genes from each positive isolate were sequenced using the bacterial primers 27f and 1492r. Basic local alignment search tool (BLAST) analysis of the sequences showed that a *Bacillus* isolate (representing OTU 2) had 99% sequence identity with *Bacillus megaterium*, a *Burkholderia* isolate (representing OTU 27) had 98% sequence identity with *Burkholderia diazotrophica* STM4206 (FN908402.1), and a *Rhizobium* isolate (representing OTU 16) had 99% sequence identity with *R. tropici* strain 233 (EU488749.1). *Bacillus megaterium* has been reported to promote plant growth (Brighigna *et al*., 1992), *Burkholderia diazotrophica* performs BNF in association with *Mimosa pudica* (Sheu *et al*., 2013), and *R. tropici* is a legume symbiont (Martinezromero *et al*., 1991).

**Figure 1:**
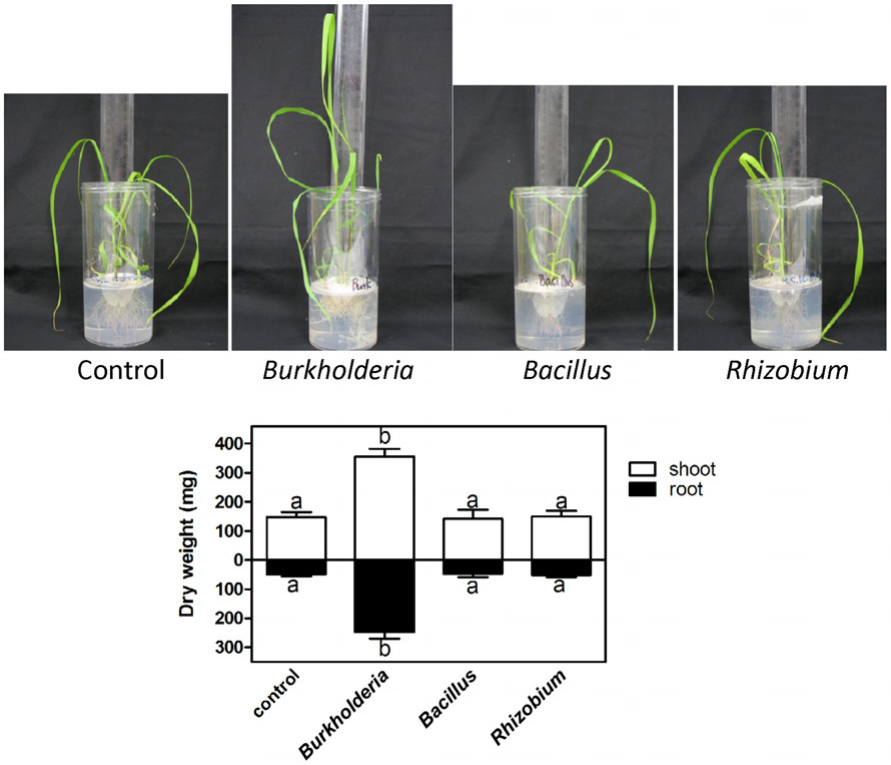
Effect of *Burkholderia*, *Bacillus* and *Rhizobium* on the growth of sugarcane variety Q208^A^. Bacterial strains were isolated from rhizosphere/root of field-grown Q208^A^ and correspond to OTUs 27, 2 and 16 respectively (Table 1). *Burkholderia* inoculum significantly increased root and shoot biomass of sugarcane plantlets whereas *Bacillus* and *Rhizobium* had no effect. Data are averages and standard errors of 10 plants in independent microcosms. Similar results were obtained in another independent experiment. Different letters indicate significant differences at *P* < 0.05 (analysis of variance, Neuman–Keuls post-hoc test).

We tested the isolates individually for their potential to promote growth of Q208^A^ plantlets in gnotobiotic conditions. Roots of axenic plantlets of Q208^A^ were inoculated with a liquid culture of each bacterial isolate, and plantlets were grown in gnotobiotic culture for 3 weeks. No growth-enhancing effect was observed with *Rhizobium* OTU 16 or *Bacillus* OTU 2. *Burkholderia* OTU 27 promoted growth of sugarcane with an increase of root and shoot biomass by 406% and 140%, respectively, compared with non-inoculated control plants (Fig. 1).

**Figure 2:**
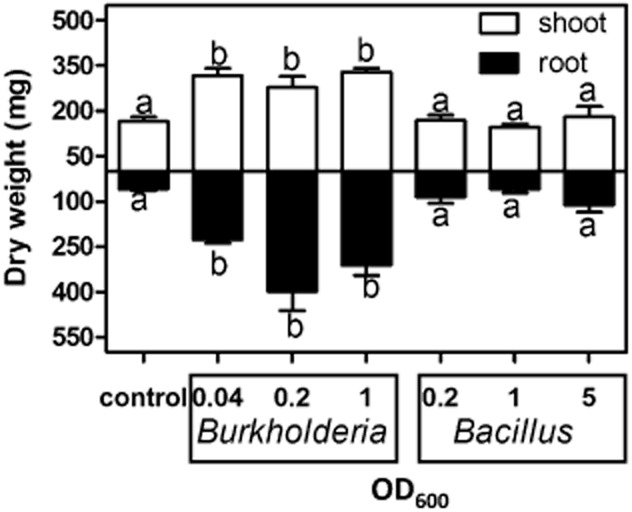
Effect of the concentrations of bacterial inoculum on the growth of sugarcane plantlets. Plants were inoculated with different ODs (OD_600_) of *Burkholderia* (representing OTU 27) and *Bacillus* (OTU 2, see Table 1) and plant dry weight was observed at 18 days post-inoculation. Control is non-inoculated axenic plants. Data represent the average and standard errors of five independent plants. Different letters indicate significant differences at *P* < 0.05, (analysis of variance, Neuman–Keuls post-hoc test).

*Burkholderia* is similar in size to *Rhizobium* (∼1 μm) but much smaller than *Bacillus* (∼10 μm). We therefore tested whether different concentrations of inoculum could be the cause of the different growth responses of plantlets. In a second experiment, we inoculated plantlets with a wide range of bacterial concentrations [*Burkholderia*: optical density (OD)_600nm_ = 0.04–1 absorbance units (corresponding dry weight: 1.5–38 mg); *Bacillus*: OD_600nm_ = 0.2–5 absorbance units (corresponding dry weight: 4.7–117 mg)]. This experiment confirmed that irrespective of bacterial concentrations, only *Burkholderia* promoted plantlet growth (Fig. 2).

The genus *Burkholderia* has emerged as an important plant-associated taxon. Sugarcane can have a high diversity of *Burkholderia* species associated with roots and stems (Boddey *et al*., 2003). In Mexico, sugarcane was associated with diazotrophic *Burkholderia uname* and *Burkholderia tropica*, and with non-diazotrophic *Burkholderia tropica* (Castro-Gonzalez *et al*., 2011). We did not detect these *Burkholderia* species in rhizosphere + roots of Q208^A^ or bulk soil, suggesting a strain-specific association between the plant host and the root microbiome, and/or specific geographical and environmental conditions selecting for particular strains. Plant growth promotion by *Burkholderia* is considered to be caused by BNF but also by solubilization of inorganic phosphate, production of siderophores and phytohormone indole-acetic acid, as well as inhibition of sugarcane pathogens *in vitro* (Luvizotto *et al*., 2010; Castro-Gonzalez *et al*., 2011). Our results confirmed previous studies showing that inoculation of *Burkholderia* enhances growth of sugarcane plants (Govindarajan *et al*., 2006).

### Identification and phenotypic characterization of the *Burkholderia* isolate

**Figure 3:**
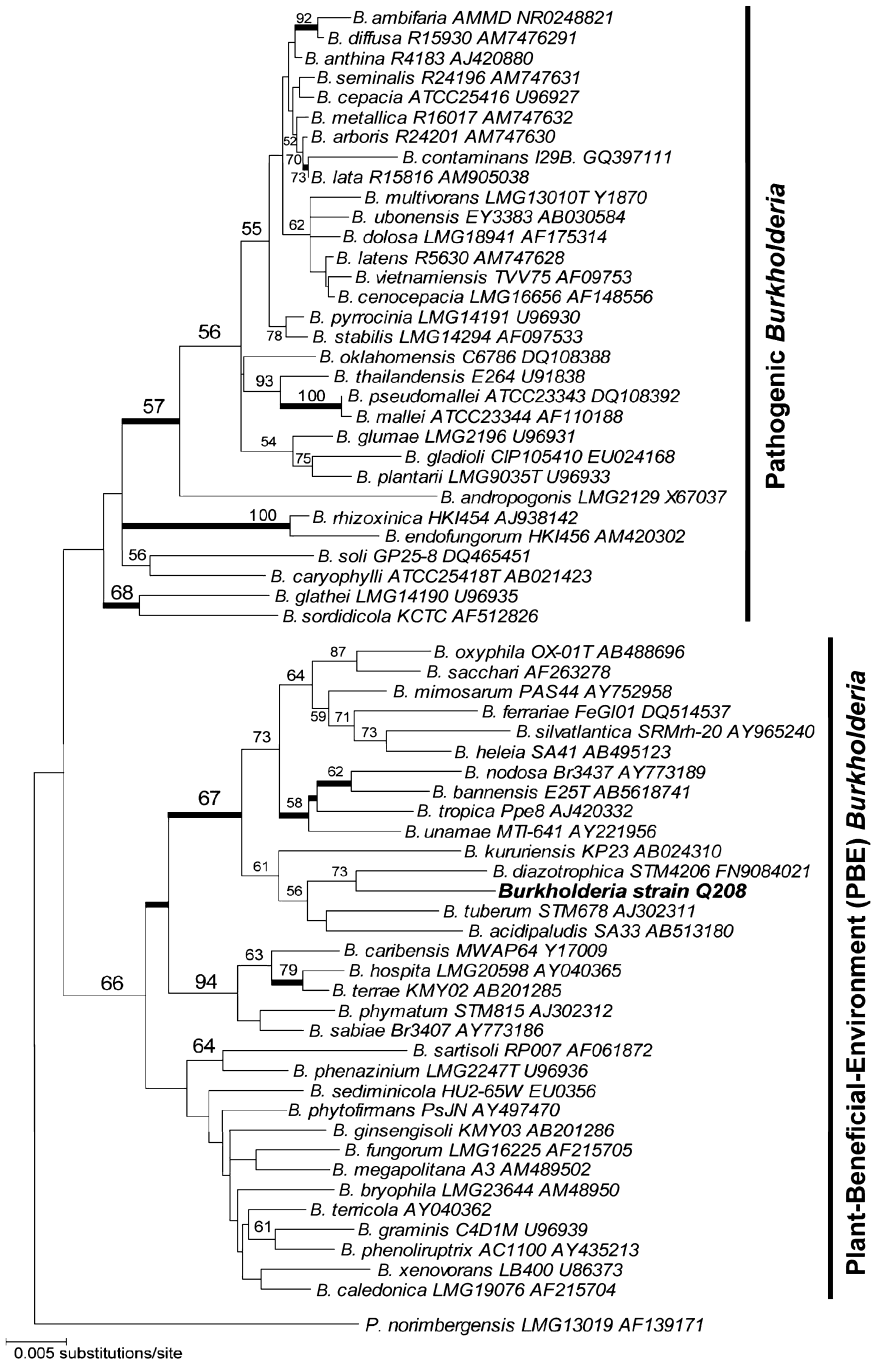
Phylogenetic tree of 16S rRNA gene sequences showing the position of *Burkholderia* strain Q208 from this study (alignment length > 1300 positions) and closely related *Burkholderia* species. The consensus tree topology was inferred using neighbour-joining with non-parametric bootstrap based on maximum composite likelihood (1000 replicates; MEGA v5.05). Bootstrap support value ≥ 50% is shown at each internal node. Thick branches indicate Bayesian posterior probabilities ≥ 0.90. Unit of branch length is in number of substitutions per site.

The closest known phylogenetic relative of the *Burkholderia* isolate (designated as strain Q208) is *Burkholderia diazotrophica* (STM4206) (Sheu *et al*., 2013) for which the recorded similarity of the 16S rRNA genes is 98% over > 1300 bp (Fig. 3).

The *Burkholderia* genus is divided into two main groups (Suárez-Moreno *et al*., 2012). The first group contains *Burkholderia* species that are pathogens in human, animals and plants. The second group includes non-pathogenic species mostly reported to be associated with and beneficial to plants. The latter group, to which *Burkholderia* strain Q208 belongs, is referred to as the ‘plant-beneficial-environment’ (PBE) *Burkholderia* group because most have useful properties as antagonists to plant pests, as PGPR, and as organisms that degrade toxic substances (Chiarini *et al*., 2006). As shown in Fig. 3, *Burkholderia* strain Q208 forms a monophyletic subclade within the PBE group on the tree together with *Burkholderia diazotrophica* STM4206 and *Burkholderia tuberum* STM678 isolated, respectively, from nodules of *Mimosa* species (Sheu *et al*., 2013) and tropical legumes (Vandamme *et al*., 2002), and from soil (e.g. *Burkholderia oxyphila* and *Burkholderia sacchari*; Bayesian posterior probability 100%; bootstrap support 67%). It should be noted that *Burkholderia vietnamensis* grouped within the pathogenic *Burkholderia* group has been reported as a PGPR (Reik *et al*., 2005). However, so far none of the bacteria from the PBE cluster have been found to be pathogenic. The position of *Burkholderia* strain Q208 in the PBE cluster makes it potentially a non-pathogenic bacterium, but this should be evaluated in more detail.

Next, we proceeded to phenotypic characterization of strain Q208 by demonstrating growth on various carbohydrates as sole carbon and energy sources (Supporting Information Appendix S1). We compared the carbon-source use profile of this strain with four closely related *Burkholderia* species in the same phylogenetic clade and three others that were isolated from non-leguminous sources. Strain Q208 can be differentiated from the type strains of other species of *Burkholderia* by its pattern of oxidation of carbon sources (Table 2). Compared with its sister taxon *Burkholderia diazotrophica* (Fig. 3), *Burkholderia* strain Q208 oxidizes fucose, N-acetyl-D-galactosamine inositol, succinic acid mono-methyl ester, acetic acid and xylitol but not adonitol, cellobiose, raffinose, sucrose or trehalose (see Sheu *et al*., 2013 for a full comparison). Contrary to *Burkholderia diazotrophica*, *Burkholderia* strain Q208 does not hydrolyse Tweens 20, 40, 60 or 80. The API 20E microtest gallery showed strain Q208 able to use citrate and to be β-galactosidase positive, but negative for nitrate reduction and urease (the latter confirmed by growth on Christensen's urease agar slope), further differentiating strain Q208 from *Burkholderia diazotrophica*.

**Table 2:** Comparison of phenotypic characteristics of strain Q208 with the type strains of related *Burkholderia* species

Characteristic	1	2	3	4	5	6	7	8
Isolation source (Reference)	Soil (Bramer *et al*., 2001)	TCE-polluted water (Zhang *et al*., 2000)	Rhizosphere (Gillis *et al*., 1995)	Plant (Aizawa *et al*., 2010)	Nodule s (Vandamme *et al*., 2002)	Nodule (Chen *et al*., 2006)	Nodule (Sheu *et al*., 2013)	Rhizosphere and soil (This study)
Nitrate reduction	+	−	*+*	+	−	*+*	*+*	−
Urease	ND	+	*+*	+	−	*+*	*+*	−
β-Galactosidase	ND	+	*+*	−	*+*	−	*+*	+
Oxidation of:								
Adonitol	+	+	−	+	+	−	+	−
Arabinose	+	+	+	+	+	+	+	+
Arabitol	+	+	+	+	+	+	+	+
Cellobiose	−	−	+	+	−	−	+	−
Fructose	+	+	+	+	+	+	+	+
Fucose	+	+	+	+	+	+	−	+
N-acetyl-D-galactosamine	+	+	+	−	−	−	−	+
Lactose	−	−	−	−	−	−	−	−
Maltose	−	+	−	−	−	−	−	−
Melibiose	−	−	−	−	−	−	−	−
Raffinose	+	−	+	−	−	−	+	−
Rhamnose	−	+	−	−	+	−	+	+
Sorbitol	+	+	+	+	+	+	+	+
Sucrose	+	−	+	−	−	−	+	−
Trehalose	−	−	+	+	−	−	+	−
Xylitol	−	+	−	−	+	−	−	+
Growth on MacConkey medium^a^	−	−	+	ND	ND	ND	ND	+
DNA G + C content (mol%)	63.7	64.8	67.9	64.0	62.8	64.8	63–65	63.3
N fixation	ND	Yes	Yes	ND	yes	Yes	Yes	Yes

a Data are from Caballero-Mellado *et al*. (2004) except for strain Q208.

Species: 1, *Burkholderia sacchari*; 2, *Burkholderia kururiensis*; 3, *Burkholderia vietnamiensis*; 4, *Burkholderia acidipaludis*; 5, *Burkholderia tuberum*; 6, *Burkholderia mimosarum*; 7, *Burkholderia diazotrophica*, 8, strain Q208.

+, Positive; −, Negative; ND, not determined; TCE, trichloroethylene.

The biochemical characteristics of strain Q208, including tested enzyme activities and carbon use profiles, show similarity to *Burkholderia tuberum* that has been isolated from root nodules of tropical legumes. The two bacteria are, however, distinguishable by their ability to oxidize adonitol and N-acetyl-D-galactosamine: strain Q208 assimilates N-acetyl-D-galactosamine, whereas *Burkholderia tuberum* does not (similar to other *Burkholderia* species isolated from nodules). In contrast with *Burkholderia tuberum*, strain Q208 does not oxidize adonitol. While strain Q208 assimilates citrate, *Burkholderia tuberum* lacks this ability (Vandamme *et al*., 2002).

Based on these phenotypic and phylogenetic analyses, we conclude that ‘strain Q208’ warrants classification as a new species in the genus *Burkholderia*, and propose the name *Burkholderia australis* sp. nov.

### Description of *Burkholderia australis* sp nov

*Burkholderia australis* [aus.tra'lis. M.L. adj. *australis* referring to Australia, where the strain was isolated]. Cells are Gram-negative, non-spore-forming and ovoids to short rods (0.4–0.5 μm in width and 1.0 ± 1.3 μm in length) and occur singly or in pairs. Growth is observed at 28, 30 and 37°C. β-Galactosidase-, catalase- and oxidase are positive. Indole is not produced, gelatin is not hydrolysed, and glucose is not fermented. Additional characteristics are listed earlier. The DNA G + C content is 63.3 mol%. The type strain is strain Q208^A^, as it was isolated from soil and rhizosphere + root of sugarcane variety Q208^A^ in Queensland, Australia.

### *Burkholderia* colonizes rhizosphere and root of sugarcane

**Figure 4:**
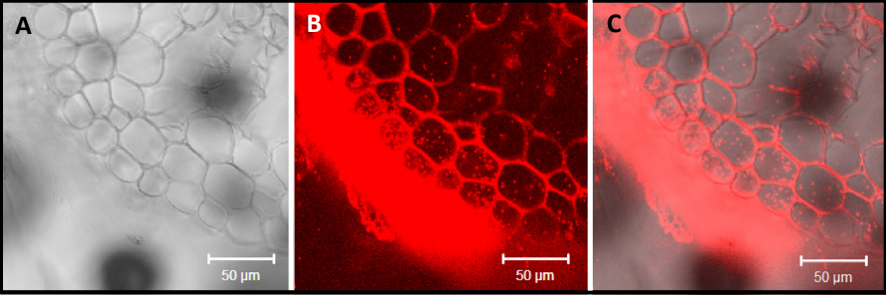
Localization of *Burkholderia australis* Q208 in roots of sugarcane tissue-culture generated plantlets. Plants were inoculated with *Burkholderia australis* and grown gnotobiotically for 5 days post inoculation. FISH showed that *Burkholderia australis* cells were abundant on the root surface and also present as endophytes in root cortex cells. (A) is the bright field image, (B) is the fluorescent image, and (C) the combined image of (A) and (B). Cytoplasmic streaming of *Burkholderia australis* on the surface and inside sugarcane root cells is presented in the supplementary movies.

Fluorescence *in situ* hybridization (FISH) using a CY3 fluorescently labelled oligonucleotide probe specific to *Burkholderia* allowed visualization of the bacterium by fluorescence confocal laser scanning microscope (CLSM). Large numbers of bacteria dwell at the surface of roots, and some occur inside root cortex cells (Fig. 4). Movement of bacterial cells with cytoplasmic streaming confirmed their localization inside living root cells (Supporting Information Movies S1 and S2). These results indicate that *Burkholderia australis* has rhizospheric and endophytic associations with sugarcane Q208^A^.

### Whole-genome sequencing of *Burkholderia australis* reveals the presence of genes involved in BNF

BNF is the enzymatic reduction of N_2_ to ammonia. Most BNF is carried out by molybdenum nitrogenase (MoFe protein containing Mo and Fe atoms), which consists of two soluble proteins NifD and NifK, and occurs in all diazotrophs (Rubio and Ludden, 2008). NifH (Fe protein) is essential in this enzymatic process as it supplies electrons to the MoFe protein. Numerous other Nif proteins are involved in the regulation of Nif activity. There are two other nitrogenases: Vnf, in which Mo in the MoFe protein is replaced by vanadium, and Anf, in which only Fe is present (Bishop and Joerger, 1990). We sequenced the genome of *Burkholderia australis* to an estimated 93% completion based on the presence of 111 bacterial single-copy marker genes (Dupont *et al*., 2012). Comparative analysis revealed that many homologs of genes involved in Nif nitrogenase are present in the genome of *Burkholderia australis* (Table 3), indicating that this isolate has the potential to perform BNF.

**Table 3:** BLAST result of nitrogenase and FeSII proteins on the genome of *Burkholderia australis*

	*Burkholderia australis* versus existing data of *Burkholderia*	
Putative protein product	% Identity	E value
NifQ (*Burkholderia multivorans* CGD2M)	49.37	3.00E−18
NifU (*Burkholderia* strain ATCC 17616 / 249)	90.3	1.00E−67
NifV (*Burkholderia phenoliruptrix* BR3459a)	32.78	1.00E−39
NifR3 (*Burkholderia* sp. YI23)	86.9	4.00E−176
NifA (*Burkholderia* sp. YI23)	71.48	0
NifQ (*Burkholderia* sp. YI23)	47.78	2.00E−38
NifH (*Burkholderia nodosa*)	47.22	5.00E−05
NifB (*Burkholderia phenoliruptrix* BR3459a)	24.26	1.00E−04
Ferredoxin, FeSII type (*Burkholderia phymatum* STM815)	93.00	1.00E−50

In addition to the presence of nitrogenase, a homolog of the FeSII protein (ferredoxin) involved in the protection of nitrogenase from irreversible inhibition by oxygen (Lery *et al*., 2010) was found (Table 3). This FeSII protein shares 93% identity (1.00E−50) with FeSII from *Burkholderia phymatum* STM815 and 43% with FeSII from *Gluconacetobacter diazotrophicus* (3e−20).

### BNF is confirmed with ^15^N_2_ detected in leaves of sugarcane plantlets

The presence of plant available N influences the success of BNF, both in association with the plant host and as free-living forms. In very N-limiting growth conditions, production of nitrogenase by N_2_ fixing bacteria is generally low, and the production of nitrogenase increases with access to available (reactive) N up to an optimal amount after which BNF is inhibited by high N availability (Reed *et al*., 2011). We investigated N concentrations suboptimal for plant growth to develop an experimental system to test whether BNF contributes to the growth-enhancing effects of *Burkholderia australis*. Axenic sugarcane plantlets were grown with ammonium-nitrate concentrations ranging from 2 to 40 mM. Plantlets grown with 20 and 40 mM ammonium nitrate had significantly (*P* < 0.05) more root and shoot biomass (Supporting Information Fig. S1) than plantlets grown with 2 or 10 mM ammonium nitrate. Because 2 mM ammonium nitrate resulted in poor overall growth of plantlets, we chose 10 mM ammonium nitrate for the next steps aimed at evaluating BNF.

**Figure 5:**
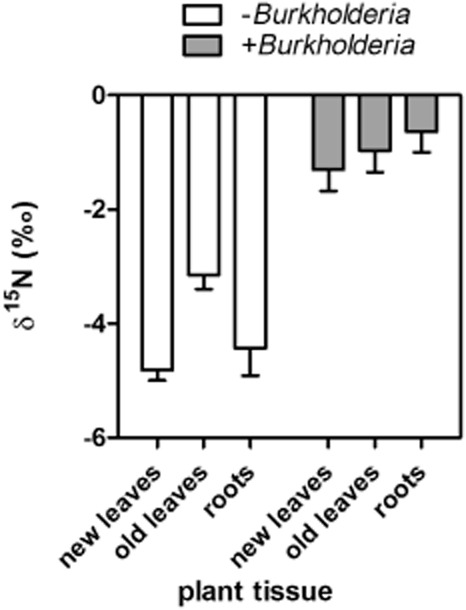
δ^15^N of sugarcane plantlets non-inoculated (control) or inoculated with *Burkholderia australis* Q208 3 days after injection with ^15^N-labelled N_2_ into growth containers. Data are means of six independent replicates and standard errors.

Sugarcane Q208^A^ plantlets grown on a medium containing 10 mM ammonium nitrate were incubated with artificial air containing ^15^N_2_ (98 atom% enrichment) in a closed vessel. After 3 days, roots and leaves were analysed for ^15^N concentrations. ^15^N of plant tissues should be enriched if BNF occurs and reactive N passed on to the plant. Roots and leaves of plants inoculated with *Burkholderia australis* were significantly (*P* < 0.05) enriched in ^15^N compared with non-inoculated plants (Fig. 5), indicating that *Burkholderia australis* is a BNF diazotroph and supply N to plantlets in the tested conditions.

However, this result does not exclude the possibility that other plant growth-promoting factors are also involved, and we have further, but not exhaustively, tested some traits. *Burkholderia australis* is unable to solubilize mineral phosphate in the form of Ca_3_(PO4)_2_ but produces siderophores when grown under iron-limiting condition (Supporting Information Fig. S2). Secreted siderophores chelate ferric ion (Fe^3+^) with high affinity, making Fe available to plant hosts and depriving a pathogen of iron (Schippers *et al*., 1987). In our study, plants were not grown under Fe-limited conditions, and the observed plant growth promotion here was unlikely to be facilitated by an increased availability of iron. However, that does not exclude the possibility that *Burkholderia australis* is involved in Fe supply in natural growth conditions (Khan *et al*., 2006).

### Conclusions

We propose that using culture-independent community profiling to identify microbial populations substantially enriched in the sugarcane rhizosphere is a useful means to guide efforts to isolate potentially interesting PGPR. Such enrichment profiling suggests that targeted isolates already form successful associations with a target host under field conditions and may have potential as biofertilizers. Such directed research represents a first step in addressing the need to improve the selection and delivery of bacteria that can contribute biologically fixed N to sugarcane crops. Subsequent research will evaluate whether the isolate identified here is specific to the studied sugarcane variety or able to colonize closely related or less closely related varieties, and how it can be delivered and form successful associations with crops in field situations.

## Experimental procedures

### Sample collection

Sampling was carried out in March 2011 at two individual plots within a 4-ha field trial (S19°43.955', E14°710.727', 26 m above sea level) near Ayr, North Queensland, Australia. The soil is a silty-clay loam. Roots with adhering soil were sampled from commercial variety Q208^A^. Five individual samples of bulk soil and roots with adhering rhizosphere were taken from each plot at 0–10 cm depth. Each sample consisted of three to five pooled subsamples. Bulk soil was free of roots and sieved to 2-mm particle size to remove larger particles. Roots with adhering soil (rhizosphere) were gently shaken to remove excess soil and constituted the rhizosphere + root samples in our study. Samples were immediately placed in a cool box for 2 days until return to the laboratory. The samples were processed immediately or stored at −20°C for isolation of bacterial isolates or DNA respectively.

### DNA extraction and pyrosequencing

Total dsDNA was extracted from 0.25 g of mixed, homogenized sieved soil or rhizosphere + root samples using Mo Bio PowerSoil DNA isolation kits following the manufacturer's instructions (Mo Bio Laboratories, Inc., Carlsbad, CA, USA). To quantify bacteria and archaea communities, the small subunit region of 16S ribosomal DNA (rDNA) gene from bulk DNA extracted from soil and rhizosphere + root samples was amplified using individually barcoded primers broadly targeting bacteria and archaea: 803F (5'-ATTAGATACCCTGGTAGTC-3') and 1392wR (5'-ACGGGCGGTGWGTRC-3') modified on the 5' end to contain the 454 FLX Titanium Lib L adapters B and A respectively. The reverse primer contained a five to six base barcode sequence positioned between the primer sequence and the adapter. A unique barcode was used for each sample. Thermocycling conditions were as follows: 95°C for 3 min; then 30 cycles of 95°C for 30 s, 55°C for 45 s, 72°C for 90 s; then 72°C for 10 min. Amplifications were performed using a VeritiH 96-well thermocycler (Applied Biosystems, Foster City, CA, USA). Amplicons were purified using a QIAquick PCR purification kit (Qiagen, Venlo, Limburg, the Netherlands), quantified using a QubitTM fluorometer with a Quant-iT dsDNA BR Assay Kit (Invitrogen, Carlsbad, CA, USA) and then normalized to 25 ng ml^−1^ and pooled for 454 pyrosequencing. Sequencing was performed by the Australian Centre for Ecogenomics at The University of Queensland. Demultiplexed sequences were run through a QIIME-based pipeline using standard methods and custom scripts (Caporaso *et al*., 2010).

### Bacterial isolation

Samples were processed by homogenizing 10 g of pooled rhizosphere + root samples from all biological replicates in a vegetable juicer (Breville, Botany, NSW, Australia) with 10 ml sterile phosphate-buffered saline, pH 7.2 for 1 min at high speed. The homogenized sample was filtered through Whatman paper number 1, and serial dilutions (up to 10^−9^) were made with the filtrate. One hundred microlitres of each dilution was spread on agar plates containing R2A minimal medium, which is suitable for growing bacteria from environmental samples (Pepper and Gerba, 2009). Plates were incubated at 28°C for 5 days. To sample *Bacillus* species, bacterial solution was heated at 80°C for 10 min before making dilutions. This treatment was used to enrich the sample in *Bacillus* endospores that are resistant to such treatment. The plates containing separated individual bacterial colonies were used for PCR screening of bacteria of interest.

### Bacteria screening by PCR

To screen for *Bacillus* (OTU 2&8), *Rhizobium* (OTU 16) and *Burkholderia* (OTU 11&27, Table 1), we used the bacterial pool of the rhizosphere + root of Q208^A^ grown on agar plates containing R2A medium (see earlier discussion). Amplification of 16S rRNA genes was carried out using PCR with specific primers (bacillus_R: TGCAGCCCTTTGTACCAT; rhizobium_R: CACACTCGCGTGCTCG; burkhold_R: GTTGGCAACCCTCTGTTC) and 926F (AAACTYAAAKGAATTGACGG). The PCR was carried out as followed: a predenaturation step for 3 min at 94°C, 10 cycles including denaturation for 30 s at 94°C, annealing for 45 s at 55°C, extension for 10 s at 72°C and another 25 cycles including denaturation for 30 s at 94°C, annealing for 45 s at 53°C, and extension for 10 s at 72°C. The PCR was terminated with a step of 10 min at 72°C. Amplicons were ∼350 bp. The PCR products were sequenced to confirm the presence of the corresponding OTU sequence. Bacteria from positive colonies were preserved in 30% glycerol at −80°C.

### Sequence of 16S rRNA genes and phylogenetic analysis

16S rRNA from isolated bacteria (*Burkholderia*, *Bacillus* and *Rhizobium*) were sequenced for species characterization. PCR amplification of 16S rRNA genes was carried out using specific primers 27f (GAGTTTGATCCTGGCTCAG) and 1492r (GGTTACCTTGTTACGACTT). PCR conditions included a predenaturation step (94°C, 3 min) followed by 35 cycles of denaturation (94°C, 30 s), annealing (55°C, 45 s) and extension (72°C, 30 s). This was followed by an elongation step (72°C, 10 min). Amplicons of nearly full-length 16S rRNA gene (∼1500 bp) were purified using QIAquick PCR purification kit (Qiagen) and sequenced (AGRF, Brisbane, Australia). The sequences were identified based on searches against GenBank nr/nt database (BLASTN). Phylogenetic tree was reconstructed using neighbour-joining (Saitou and Nei, 1987) as implemented in MEGA 5.05 (Tamura *et al*., 2011); bootstrap support (Felsenstein, 1985) were generated based on 1000 replicates. In parallel, we adopted a Bayesian phylogenetic approach on the same data set using MRBAYES 3.2 (Ronquist *et al*., 2012) (MCMC ngen = 5 000 000 generations, samplefreq = 100, burn-in = 20000 samples, nchain = 4).

#### Nucleotide sequence accession numbers

16S rRNA gene amplicon pyrosequencing and *Burkholderia* genome data were deposited in GeneBank (SRP029963 and SRP029967 respectively). The complete 16S rDNA sequence of *Burkholderia australis* was deposited in GenBank (BankIt1604857 Seq1 KC608183).

### Whole-genome sequencing and genes involved in BNF

*DNA library preparation:* dual-indexed paired-end DNA libraries were prepared using genomic DNA isolated from a pure culture of *Burkholderia australis*. Fifty nanograms of DNA was fragmented and tagged with unique adapter sequences using a Nextera™ DNA Sample Preparation Kit (Illumina, San Diego, CA, USA). Index sequences were added to ends of DNA fragments via a limited-cycle PCR. The library was sequenced on an Illumina HiSeq 2000 platform at the Institute for Molecular Bioscience, The University of Queensland. Upon removal of adapter sequences and quality filtering (90% of reads having base quality score ≥ 35), the reads were assembled *de novo* using the CLC Genomics Workbench version 5.5 (CLC bio, Aarhus, Denmark) at default settings. Completeness of the assembly was assessed by the presence or absence of 111 bacterial single-copy genes (Dupont *et al*., 2012). Publicly available nitrogenase and FeSII amino acid sequences of *Burkholderia* were used as query to scan the *Burkholderia australis* genome sequence. Sequence similarity searches were performed using BLAST (Altschul *et al*., 1990).

### Morphological, physiological and biochemical characterization

Phenotypic characterization of the *Burkholderia australis* were carried out according to standard protocols (Gerhardt *et al*., 1994) using cells grown on nutrient agar at 28°C for 2 days. Morphological studies were performed with a light microscope (Eclipse E600, Nikon, Tokyo, Japan) equipped with a SPOT digital camera (SPOT™ Diagnostic Instrument, Inc., Sterling Heights, MI, USA). Gram staining was performed using the Hucker method (Murray *et al*., 1994). Physiological and biochemical characteristics were tested using the API 20E system (bioMérieux, Craponne, France) according to the manufacturer's instructions. To test carbon substrate assimilation, GN2 microtitre test plates were used (Biolog, Hayward, CA, USA). Early exponential-phase cultures were used as inocula for the test plate (150 μl per well). Plates were incubated at 28°C and examined after 24 and 48 h to allow development of colour indicative of substrate oxidation. In addition, phenotypic features were analysed by growing the strain on MacConkey agar plates and Christensen's urease agar slope (Smibert and Krieg, 1994). DNA G + C content was analysed by whole-genome sequencing (details earlier).

### Putative ability of BNF, phosphate solubilization and production of siderophores

To evaluate the putative ability of BNF of isolates, single colonies were inoculated on Burk's N-free medium (Burk and Lineweaver, 1930) [0.8 g K_2_HPO_4_, 0.2 g KH_2_PO_4_ (pH 7.3); 0.2 g MgSO_4_; 0.2 g NaCl; 0.1 g CaSO_4_; 0.01 g Fe_2_(SO_4_)_3_; 10 g glucose; 1000 ml water]. The plates were incubated at 28°C for 5 days.

Phosphate solubilization was tested using National Botanical Research Institute's phosphate growth medium (Nautiyal, 1999). Phosphate-solubilizing activity was detected by the presence of a translucent halo around the colony, which was measured after 3 days of growth at 28°C.

Siderophores production was detected by using the Chrome azurol S (CAS) agar assay (Schwyn and Neilands, 1987). Siderophores production was indicated by orange halos around the colonies after the incubation at 28°C for 3 days.

### Plant growth conditions

Seedlings of cultivar Q208^A^ were micropropagated. Apical meristems were grown on Petri dishes containing Murashige and Skoog (MS, Murashige and Skoog, 1962) medium solidified using 3.2 g l^−1^ phytagel (Sigma-Aldrich, St. Louis, MO, USA) at 28°C, light intensity at 400 μmol m^−2^ s^−1^ with a 16-8 h day-night regime. The fully developed seedlings were obtained after 3 months with several subcultivations. Uniform fully developed seedlings with 2–3 cm long roots were used for the inoculation experiments.

### Bacterial inoculation of plants

Bacteria were grown to mid-log phase in a nutrient broth medium (Sigma-Aldrich), centrifuged at 4500 *g* for 15 min, washed twice and suspended in sterile water. Bacterial solutions at optical density of 1 (approximately 10^9^ cell ml^−1^) were used for inoculation. Roots of sugarcane plantlets were dipped in bacterial solution for 15 min before being transferred on half-strength MS media (10 mM ammonium nitrate) supplemented with 20 g sucrose and 3.2 g l^−1^ phytagel (Sigma-Aldrich) with pH adjusted to 5.7. Plants were kept in a growth cabinet (28°C, 16 h/8 h day/night, 400 μmol m^−2^ s^−1^) for 18 days after inoculation.

### ^15^N_2_ experiment to assess BNF

Six axenic sugarcane Q208^A^ plantlets were inoculated with *Burkholderia australis* as described earlier and grown for 7 days in 50 ml tubes with cotton wool cap. A further six non-inoculated plants served as control. The tubes were then sealed with rubber seals and 10% of the headspace was replaced with ^15^N_2_ (98 atom % ^15^N, Sigma-Aldrich). The tubes were returned to the growth chamber, and after 3 days of incubation (i.e., 10 days post inoculation, dpi), plants were harvested. Plants were separated into roots, young green leaves and old yellow leaves, and rinsed three times in 0.5 mM CaCl_2_. Samples were dried at 60°C for 3 days, weighed, homogenized and analysed for ^15^N content with continuous-flow isotope ratio mass spectrometer (Stable Isotope Facility, University of California, Davis, CA, USA). The δ^15^N value is calculated as [R15(sample)/R15(standard) − 1] × 1000 where R15 = ^15^N/^14^N of the sample, and the standard is air R15(air) = 0.0036765 (Fry, 2006).

### Microscopy analysis

CLSM analysis: the middle parts of primary roots regions (∼15 mm long) were washed and embedded in 3% agarose and sectioned with vibratome (Leica VT 1200S, Leica, Wetzlar, Germany). Root were coated with agarose before processing to ensure that bacteria external to roots were trapped in the agarose and not dislodged during cutting. Sections were transferred into curved slides, washed thoroughly with deionized water, and analysed by CLSM or further processed by FISH.

FISH analysis: sugarcane roots sections embedded in agarose (see earlier discussion) were fixed in 4% paraformaldehyde and treated with oligonucleotide probe specific to *Burkholderia* [Burkho–CY3 ACCCTCTGTTCCGACCAT (Hogardt *et al*., 2000)] following methods described by (Manz *et al*., 1992). FISH slides were mounted with Citifluor, to avoid bleaching, and a Zeiss LSM510 META CLSM (Carl Zeiss, Jena, Germany) was used with 10 × dry, 20 × water immersion objectives, 40 × and 60 × oil immersion objectives. Cy3-labelled *Burkholderia* probe was visualized by excitation with HeNe1 laser at 543 nm; detection with a 560–615 nm band-path filter.

## Supporting information

**Fig. S1.** Effect of nitrogen concentrations in MS growth medium on the growth of sugarcane Q208^A^. Data are averages and standard errors of three plants in independent microcosms. **Fig. S2.** Growth of *Burkholderia australis* on selective media. *Burkholderia australis* (indicated by red asterisk) was able to grow on N-free medium (A) and to produce siderophore (B), but it was not able to solubilize mineral phosphate (C). Other isolates were used as positive and negative controls. **Movie S1 and S2.** Cytoplasmic streaming of *Burkholderia* *australis* on the surface and inside sugarcane root cells. Axenic sugarcane plantlets were inoculated with *Burkholderia australis* and grown gnotobiotically for 5 days post-inoculation (see Experimental procedures for details). The movie was taken with a confocal microscope (Zeiss LSM501 Meta). **Appendix S1.** Strain Q208 showed growth on various types of carbohydrate as the sole carbon and energy source. When the Biolog GN2 microlitre test system was used, the following substrates were oxidized: N-acetyl-Dglucosamine, L-arabinose, D-arabitol, D-fructose, L-fucose, D-galactose, α-D-glucose, m-inositol, D-mannitol, D-mannose, L-rhamnose, D-sorbitol, xylitol, pyruvic acid methyl ester, succinic acid monomethyl ester, acetic acid, *cis*-aconitic acid, formic acid, D-gluconic acid, α/γ-hydroxybutyric acid, itaconic acid, α-ketobutyric acid, D,Llactic acid, propionic acid, succinic acid, bromosuccinic acid, L-alaninamide, D,L-alanine, L-asparagine, L-aspartic acid, L-glutamic acid, L-histidine, hydroxy-L-proline, L-leucine, L-proline, γ-aminobutyric acid, and D-glucose-6-phosphate.
